# Physical Activity among Adults with Low Socioeconomic Status Living in Industrialized Countries: A Meta-Ethnographic Approach to Understanding Socioecological Complexities

**DOI:** 10.1155/2020/4283027

**Published:** 2020-04-01

**Authors:** Lal B. Rawal, Ben J. Smith, Henry Quach, Andre M. N. Renzaho

**Affiliations:** ^1^School of Health, Medical and Applied Sciences, Central Queensland University, Sydney Campus, Sydney, Australia; ^2^School of Social Sciences, Western Sydney University, Sydney, Australia; ^3^Translational Health Research Institute, Western Sydney University, Sydney, Australia; ^4^School of Public Health, University of Sydney, Sydney, Australia; ^5^School of Public Health and Preventive Medicine, Monash University, Melbourne, Australia

## Abstract

**Method:**

Using MeSH keywords, we searched major electronic databases including Medline, EMBASE, CINAHL, and PsycINFO in order to identify relevant publications published between January 2000 and October 2018. We included 19 qualitative studies which met inclusion criteria and were focused on physical activity determinants among adults.

**Results:**

Determinants emerging from these studies were grouped into six themes: (i) urban environment, (ii) financial constraints, (iii) work-life integration, (iv) community engagement, (v) social support, and (vi) psychosocial factors. After conceptualising these six themes into a social ecological model, we identified potential research gaps for physical activity among adults with low socioeconomic status living in industrialized countries.

**Conclusion:**

Our major insight was that, in industrialized countries, physical activity overlooks potential strengths to maintain health and well-being of those people with low socioeconomic status. A more complex understanding of contradictions between positive and deficit frames would lead to more critical insights of research gaps of physical activity in adult population with low socioeconomic status.

## 1. Introduction

Physical inactivity is increasingly recognized as one of the leading causes of mortality worldwide [1–3]. Evidence shows that, annually over 5 million people worldwide die due to low or insufficient physical activity (PA), which accounts for 6% of global deaths [3, 4]. Insufficient PA can impact considerably on health and productivity and is a cause of many chronic diseases such as coronary heart disease, type 2 diabetes, breast cancer, and colon cancer [1, 3, 5, 6]. Regular PA is a protective factor not only for leading chronic diseases [4] but for a range of important disease risk factors such as hypertension, overweight, and obesity [7]. Recently published evidence shows that there is still over a quarter of the global population who do not undertake recommended levels of PA, and this proportion is higher among women than men [8]. In 2018, the World Health Organization launched its' Global Action Plan on Physical Activity (GAPPA) 2018–2030 emphasizing “More people active for a healthier world,” which aims to achieve reduction of 15% in physical inactivity levels by 2030 [1]. The targets and policy recommendations of GAPPA are envisioned to contribute to the achievement of a number of Sustainable Development Goals (SDGs) [9].

PA varies across sociodemographic groups; not all adults have equal access to PA opportunities as part of their daily lifestyle [10–12]. People of low socioeconomic status are more likely to have poorer health and shorter life expectancy than people of higher socioeconomic status [10, 12], attributed in part to a lower prevalence of PA [13]. Models for PA promotion identify individual factors such as age, sex, health status, self-efficacy, and motivation [14, 15]. Health promotion models also recognise the role of contextual influences and the effect of interactions between individual, social, and physical environmental factors [16–18]. The relevance of these models extends to mass media campaigns for PA, where message design processes need to be supported by environmental and policy actions that enable and reinforce the adoption of behaviour change [19].

Whilst the literature commonly focuses on socioeconomic status related disparities in PA [13, 20, 21], it should be acknowledged that culture, gender, disability, and a host of other sociodemographic factors intersect to impact movement opportunities in different contexts [22]. Therefore, the focus on the term “socioeconomic” in the current literature, particularly when it is defined by rigid economic measures, may have excluded relevant studies. The intent of this investigation was to explore broadly, across contexts, how qualitative literature has elucidated the relationships between socioeconomic disadvantage and PA.

The Social Ecological Model (SEM) has been well recognized worldwide and been used broadly in health sectors [23–25] including for the improvement of PA among a range of populations and in different settings [23, 26–29]. The SEM emphasizes understanding the multifaceted and interactive effects of multiple factors, including those that are personal and environmental, upon behaviour [18, 23, 30]. Studies have emphasized the need for understanding and social and contextual correlates in order to ensure appropriate use of SEM for achieving PA outcomes [23, 26–29]. However, the SEM has been critiqued for a lack of “*sufficient specificity to guide conceptualisation of specific problems or to identify appropriate interventions*” [31]. Bauman et al. argued that SEM can evolve to become more context specific when evidence generation is planned for the purpose of creating more focused structural models [14, 15]. When the SEM becomes context specific, it can better “*expose and account for complexity of sociocultural and environmental effects*” to guide coordinated interventions [32]. The disparities in PA across the sociodemographic spectrum have been explored using both quantitative and qualitative research methods. Meta-analyses of quantitative studies concerning PA within low socioeconomic groups have provided important insights to further explore this phenomenon [13, 33].

Evidence suggests that disparities exist across personal, social, and environmental determinants of PA, which contribute to social disparities in the achievement of PA targets [20, 34]. The available evidence provides a compelling case for paying close attention to socioeconomic and cultural disparities in formulating policies and developing intervention approaches for PA promotion [2, 35, 36]. To explain PA inequalities between socioeconomic groups, SEM can take an account of the socioeconomic and cultural backgrounds of population subgroups with regards to their decision-making [37].

A number of philosophical approaches to synthesizing the qualitative data have been in practice [38, 39]. Some are based on analysis methods used in primary research, and most use either integrative or interpretative approach to synthesizing qualitative evidence base [38, 39]. Meta-ethnography is an interpretive approach originally developed by Noblit and Hare [40], and data from primary studies are synthesised to achieve new conceptual understandings or produce new models or theories [41]. The synthesis approach has the potential to advance level analyses to generate new evidence base, find a new research question, and reduce duplication in research [42, 43]. This approach has been used in a range of sectors including public health [44, 45]. Meta-ethnography is an opportunity for qualitative research to expand on theoretical approaches to the promotion of PA involving individual, social, and environmental influences [17, 46]. To this end, our philosophical approach was interpretative in nature and sought to be objective and systematic by applying key criteria to ensure reliability and validity of our interpretive inferences [47, 48].

The aim of this study was to undertake a meta-ethnographic analysis of qualitative studies to explore complex social ecological aspects of PA among socioeconomically disadvantaged groups living in industrialized countries. We explored and summarised the breadth of qualitative findings across contexts, to capture an overarching social ecological account of what qualitative research have determined in relation to socioecological determinants and to use this to highlight strengths and gaps in PA research. The intent was for future research to benefit from a broad summative understanding of what is known qualitatively about individual, social, and environmental influences on PA across complex societal systems (cultures, countries, ages, and settings).

## 2. Methods

### 2.1. Study Design

In this study, we adopted Noblit and Hare's seven-step approach to synthesise findings from qualitative studies into a social ecological frame [40]. The seven steps included: getting started; deciding what is relevant to the initial interest; reading the studies; determining how the studies are related; translating the studies into one another; synthesizing translations, and expressing the synthesis. We categorized the seven-step process into three major stages including (i) selecting studies, (ii) synthesizing translations, and (iii) presenting the synthesis. Firstly, we identified the research interest and selected articles that were closely related to our initial interest. Secondly, we assembled all the studies together and determined how they are related to each other. We then translated the studies with each other based on a comparative approach. In addition, we determined and synthesized the commonalities and differences between each account and derived a new framework that not only maintains the central concept of individual interpretation but also reveals a more comprehensive explanation in comparison to what each part alone implies. Thirdly, we presented our new metaphor (i.e., proposed social ecological model for PA) in the form of a diagram to facilitate understanding of PA among the disadvantaged population groups.

### 2.2. Search of Relevant Literature and Study Selection

We conducted searches of four major online databases including Medline, EMBASE, CINAHL, and PsycINFO using MeSH keywords for Medline and CINAHL, subject headings for EMBASE, and a standard keyword search for all four databases. The key search words and their combination were based on the study's aim. MeSH keywords and subject headings used search terms based on “physical activity,” “determinants,” and “socioeconomic status.” The final search was as follows: [[“Physical activity” [MeSH/Subject heading] and “Determinants” [MeSH/Subject heading] and “Socioeconomic status” [MeSH/Subject heading] and “qualitative*∗*”] or [“Physical activity” [Standard keyword] and “Determinants” [Standard keyword] and “Socioeconomic status” [Standard keyword] and “qualitative*∗*”]] for each individual database. As MeSH and subject heading words change over time, replication of the study should commit to the three concepts “Physical Activity,” “Determinants,” and “Socioeconomic status” when selecting various MeSH and subject headings.

### 2.3. Inclusion/Exclusion Criteria and Study Selection

Studies were included in the review based on the following criteria: (i) they focused on PA among socioeconomic disadvantaged communities, (ii) were qualitative in design, (iii) focused on adults (>18 years but <65 years), (iv) were published in English, and (v) were published between January 2000 through October 2018. The decision to include or exclude a study was made using an EndNote database in a three-phase process after removing duplicates: first reading the title, then the abstract, and finally the full text. Additionally, references for each of the studies were screened manually to identify any additional articles that were not detected by our database searches.

### 2.4. Screening Process and Data Extraction

The initial screening of articles was undertaken by HQ, and final check was done by LR. The list of references of the selected articles was manually checked for any missing articles. The data were independently extracted by HQ and were final checked by LR using a piloted form that included research aim, authors' details, year of publication, country, sampling method, sample size, population ethnicity, data collection methodology, health topics discussed in addition to PA, views expressed by study respondents (first-order constructs), the interpretations of these views by study authors (second-order constructs), and limitations.

### 2.5. Thematic Analysis

The process for thematic analysis was derived from the study by Malpass et al. [49]. Both reviewers (HQ and LR) read twice over the articles chronologically to identify emerging themes. The identified themes represented commonalities between study authors' interpretations of relevant data across individual articles. A data extraction form was developed to record the views expressed by study respondents (first-order constructs) and the interpretations of these views by study authors (second-order constructs) for each individual article. The reviewers then independently consolidated the first- and second-order constructs of the individual articles into a summary definition (translation) to produce a third-order construct. Following this, reviewers compared their summary definitions and their respective second-order constructs and worked collaboratively to synthesise them into third-order constructs.

The constructs were further reviewed by members of the research team (LR, BS, and AR) to provide new perspectives on third-order constructs. This process facilitated a consensus about a “line of argument,” a technique to synthesise translations [44]. The reviewers used third-order labels—labels are names of a group of constructs—as suggested frames for crafting a “line of argument.” The “line of argument” explains the rationale behind the suggested third-order label; different reviewers will craft different variations of a “line of argument.” Suggested third-order labels were located within one of the individual, microsystem, mesosystem, exosystem, or macrosystem layers. The key articles most influential in the construct of the summary definition of the first- and second-order constructs are bolded in Table 1.

### 2.6. Critical Appraisal

The quality of the studies included in the review was assessed using a checklist for assessing reporting standards of qualitative studies, derived from the “Consolidated Criteria for Reporting Qualitative Research (COREQ)” [50]. Nineteen checklist items relevant to the quality assessment, particularly for this study purpose, were selected as dimensions to form the criteria. Examples included (1) discussion of reflexivity, (2) statement of methodological theory that underpinned the study, and (3) reporting how participants were selected. The dimensions were scored as 0 if not present and 1 if present. The reviewers cross checked their independent assessments before reaching consensus on reporting standard scores. The maximum quality scores sum up to 19 points, and based on the discussion within authors and agreement up on, the quality levels were categorised into three groups: high (14–19); medium (7–13); and low (0–6) (Table 2). Effort was made to assess the quality of the studies in terms of how researchers have presented their thoughts and understanding to the topic based on their critical reflection of the subjective observation.

The qualitative data processing and analyses presented in this study adhered to the Standards for Reporting Qualitative Research (SRQR) [51], and strategies were employed to enhance the trustworthiness (credibility, transferability, dependability, confirmability, and transferability) of the study findings [52, 53]. This included checking the data for accuracy, organising meetings for completeness of information (HQ and LR), using team meeting for inclusion/exclusion consensus, and providing adequate information about the studies included (see Appendix-A, SRQR checklist as a supplementary document).

## 3. Results and Discussion

### 3.1. Description of Studies

Nineteen articles were included for synthesis. Their characteristics are shown in Table 3. Eleven studies were conducted in the United States, three in the United Kingdom, two in Australia, two in the Netherlands, and one in Canada. Fourteen studies used focus groups, and five used in-depth interviews. The age of participants ranged from 14–89 years. The age range of participants in some studies was not clearly defined and included adolescents and elderly people, as well as adults.

### 3.2. Description of Themes

**Figure 1:**
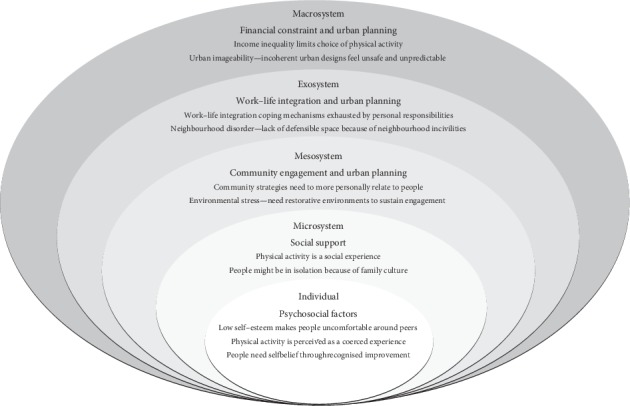
Proposed social ecological model of physical activity determinants in low socioeconomic contexts.

Our metasynthesis of first- and second-order constructs generated twelve third-order constructs and six primary themes/third-order labels (Table 1). The primary themes have been organised within a social ecological model that is shown in Figure 1. We ordered the primary themes from broadest (macrosystem) to narrowest (individual) in the description to help visualise authors' analysis.

### 3.3. Urban Environment: Macrosystem to Mesosystem

Participation in PA is influenced by accessibility to resources that support PA. People desire to travel to parks, public sports centres, or local private health clubs to pursue PA but lack access to reliable transportation such as private cars, public transport, and bicycle infrastructure [54, 56, 57, 60, 69]. In addition, the availability of peers in close proximity and the location influences walking and other forms of PA for transport [63]. If parents have the means to reach PA facilities, they would need more convenient opening times that take into account working hours and make childcare services accessible [69]. The recurring theme of lack of reliable transportation and transport facilities within close proximity may arise from urban planning.

The effect of poor urban planning is further compounded by the aesthetic feel of the neighbourhood environment. People consistently reported being exposed to feelings of neglect and depression [54, 56, 59, 61, 70, 71]. In one study, mothers reported being unable to overlook the poor state of the environment or escape the sadness of living in a place that has seemingly been left to decline [54]. Other studies highlight the importance of well-maintained paths [55, 59, 66, 71, 72], such as sidewalks with sufficient streetlights and properly paved and resurfaced roads [59]. This, along with parks, make the neighbourhood look green and inviting [61] rather than vandalised and decaying [66].

The suitability of a place for PA also takes into account perceptions of neighbourhood disorder, particularly in relation to social crime and violence. Analysis reveals that the perceived crime rate of a community can significantly affect how people use community resources for recreation. Issues related to crime and violence as reported, included the prevalence of drug use [56, 58, 59, 65, 69], police presence [58, 59, 66], muggings and homicide [59], gangs, bullying and abduction by strangers [65, 70, 71] and gunshots, and vandalism [66], especially within dark unlit areas [61, 66, 69]. Noncriminal environmental stressors in the neighbourhood included accounts of traffic-related issues such as dangerous roads and careless drivers [54, 59, 65, 66], stray dogs [55, 59, 66], poor street lighting [55–57, 59], homes being situated on highways and lack of sidewalks [57], broken glass on the ground [65], and the presence of dirty needles and cigarettes [69].

### 3.4. Financial Constraints: Macrosystem

PA participation is often affected by whether people can afford the fees and resources required. Sports equipment and sports club contributions are considered high expenditures, especially by single mothers and people who rely on social payments for day-to-day living [61]. Refreshments [64] and childcare services [64, 65, 69] are further costs incurred by parents. Parents report having to prioritise spending on their children for sports [64, 69], which means forgoing expenditure on themselves [65]. As such, demand for low-cost sports facilities and gymnasiums is evident across multiple studies [61, 64, 65, 71] with one study highlighting that cost is the number one cause of physical inactivity for a number of people [61]. There is recognition of the potential value of low-cost PA facilities such as subsidised gyms [56, 64, 69–71] and opportunity to walk and run in the local area [64]. However, the complexities of discounted fees were revealed with the finding that even reduced cost membership contracts may be too constraining to access the PA services [64].

### 3.5. Work-Life Integration: Exosystem

PA participation can be affected by the nature of the time constraints upon socially disadvantaged adults. Inflexible work hours [56, 63, 66, 67, 72] and constant family responsibilities [64, 66, 70] are major factors that also contribute to feelings of exhaustion [56, 62, 69]. Put simply, time and energy expenditures are prioritised for work, school, and family, and PA is perceived as an additional burden. It is evident that there are social differences in the scope that individuals have to balance work-life demands and that coping mechanisms are exhausted by personal responsibilities.

### 3.6. Community Engagement: Mesosystem

PA interventions have had limited impact due to poor design. It has been reported that conventional marketing of PA programs has been hindered by delivery in a narrow range of languages and with limited resources devoted to communicating about local activities [64, 69, 71]. Although lack of resources for multilingual services may prevent tailoring PA activities effectively [66], friendly nonprejudicial and socialisation environments can build trust within the communities [69]. This highlights that community engagement strategies should be tailored to meet the needs of culturally diverse groups.

### 3.7. Social Support: Microsystem

PA is heavily influenced by encouragement from a person's main sources of social support such as friends, parents, family members, coaches, and health professionals [63]. However, lacking an exercise companion is not always a barrier to PA because some people prefer to exercise alone [56, 70]. People have mixed opinions about exercising with co-workers during workplace funded fitness programs [64] further complicating interpersonal relationships in group fitness programs and how they affect the social experience. Evidently, social support from community networks is a motivator to engage in PA because of the encouragement and sense of security that it provides [59].

It is reported that PA can be influenced by negative social experiences. Some people can be made to feel guilty by family members who consider PA to be a selfish and low-priority activity [56, 69]. There can even be weight-related teasing [69], social misconceptions, and cultural stigma within and between families [65, 66, 71]. This suggests the possibility that people are isolated from physical activities because of family culture.

### 3.8. Psychosocial Factors: Individual

PA can be affected by poor self-image. Some people feel flabby in physical appearance [55] and have preconceived notions that people in fitness clubs are mostly slim and physically fit [64]. Body image issues also mean difficulty finding appropriate clothing to wear in public [69–71]. Self-esteem can determine how people feel around physically fit peers. For some, transport-related walking feels like a chore and is described as a negative experience that is burdensome and unnecessary [54]. This is compounded by perceived low levels of ability to participate in certain types of exercises because of advancing age, chronic health conditions [55], physical disabilities, poor mental health [66], fatigue, physical discomfort, or low fitness levels [62, 63]. Interestingly, people refer to boredom and stress and being “stuck in a rut” or simply feeling embarrassed rather than refer to psychological barriers as issues of mental health [65]. Some studies highlight that some people perceive themselves as having low physical competence because of previous experiences with PA. One study discusses the concept of “mastery experiences” meaning that successes are associated with ongoing participation [55]. Conversely, negative experiences discourage participation [55, 56, 70, 71]. This suggests that people need self-belief through recognised improvement in performance.

## 4. Implications for Future Research

This study employed meta-ethnography to highlight how understandings of PA participation in low socioeconomic status populations have been reported in the literature. Findings reveal how broader themes (macrosystem) have more sources than narrower themes (individual), and this emphasizes the needs for more research focused on individual-level influences on PA in socioeconomically disadvantaged communities.

Across all nineteen studies included in this review, we were unable to discover any evidence of the perceived value of PA as a positive social construct in socioeconomically disadvantaged communities. We discovered that the literature has focused on the barriers for PA rather than what supports PA among the socioeconomically disadvantaged populations. However, the literature has shifted to positively framed asset-based approaches to promoting health [73, 74].

It appears important for the future promotion of PA to consider how resources are made available and promoted to target communities. For example, qualitative research may benefit from exploring informal and less-structured forms of PA. Furthermore, qualitative research can explore seemingly unconventional activities to examine the extent to which these can be recognised as offering viable sources of PA. Future meta-ethnographies may benefit from research that can adopt a salutogenic lens [73, 74] to deliver more positive insights about what people in socioeconomically disadvantaged communities do well to sustain PA. This would require a sense of coherence research orientation that probes the necessary life experiences needed to confront stressors on healthy living [75], believing that the challenge to cope with stressors are understood (comprehensibility), believing that the resources to cope with stressors are available (manageability), and wishing to be motivated to cope with stressors (meaningfulness), removing the focus on only PA barriers as influencing PA behaviour.

## 5. Limitations

In line with the aim of this study, we summarise what is known qualitatively about individual, social, and environmental influences upon PA within and across complex societal systems. This meta-ethnography therefore used a broad-brush approach to compare commonalties across selected socioeconomically disadvantaged population groups. Because of this, the findings of this study may not be grounded or contextually relevant to all population groups. The search criteria limiting the results to qualitative studies may have excluded relevant studies without the word qualitative as a keyword. Additionally, socioeconomic disadvantage is not readily defined, nor is distinct from other forms of disadvantage or exclusion [22]. We recommend future meta-ethnographies explore more broadly concepts that intersect to form class structures. Nonetheless, our findings are useful for future qualitative researchers to hypothesise new research directions based on where they see hidden complexities and intersectional insights.

## 6. Conclusion

In this study, we propose a social ecological framework by conducting a meta-ethnography of PA determinants in low socioeconomic status communities residing in industrialized countries. Future qualitative researchers can use findings from this meta-ethnography to theorise the potential complexities and intersections that would illuminate the interconnectedness of influences on PA in people with low socioeconomic status. We believe that a more complex understanding of contradictions between positive and deficit frames would lead to more critical insights concerning research gaps of PA in low socioeconomic status.

## Figures and Tables

**Table 1:** Translation of 1st and 2nd order constructs and interpretation through 3rd order constructs.

3^rd^ order labels	3^rd^ order constructs	2^nd^ order constructs	Summary definition (translation) of the 1^st^ and 2^nd^ order constructs	Sources
Urban planning	Poor urban planning leads to inaccessibility of resources	Inaccessibility of resources	Lack of reliable transportation restricts access to neighbourhood recreational facilities, especially for those who live in remote areas. Lack of childcare and inconvenient hours of operation are policy barriers to entry	1, 3, **4**, 6, 7, 10, **12**, 13, 14, **16,** 17, 18

	Poor urban design and maintenance contributes to negative feelings	Poor neighbourhood aesthetic qualities	Poor neighbourhood appearances are a disincentive to being active. Poor environment because of poorly maintained footpaths, parks, and roads, in addition to vandalism are visually depressing and make people feel neglected	**1**, 2, 3, 4, 5, **6**, 7, 8, 13, 17, 19

	People need to feel protected in environment	Fear of crime and violence	Threatening situations such as gunshots, vandalism, drug trafficking, muggings, theft, prostitution, and homicide are some reasons why people want more law enforcement and prefer to stay home than exercise outside or even the gym. Threats from gangs, older children, and fear of abduction are on the minds of parents	2, 3, **5**, **6**, 8, 12, 13, 15, 16, 17, 19

		Neighbourhood safety hazards	Opportunities for PA are influenced by perceived safety or danger of neighbourhood, i.e., traffic, unleashed dogs, poor lighting at night, homes located on highways, decaying footpaths, people in parks doing drugs, pranks, and obscenities; glass on the floor, dirty needles, and cigarettes are especially concerning for parents	**1**, 2, 3, 4, 5, **6**, **12**, 13, 16, 17, 19

Financial constraint	Income inequality because of limited choice for PA	Affordability	Cost is regarded as a major barrier with equipment and sports clubs' contributions perceived as too expensive. Expensive drinks after exercise add to increased costs. Childcare and transportation costs also financial barriers	8, **11**, 12, 13, 14, **16,** 19

		Recognition of low-cost physical activity alternatives	Cheaper alternatives for PA are recognised such as subsidies at gyms for low-income people or just running outside	**3**, **11**, 16, 17

		Inflexible, long-term commitment and unaffordable options	Pricing strategies welcomed but commitments with lengthy memberships can deter people from participating in sports. Perceived stigma for being below poverty line when requesting subsidies also reduces participation	**11**, 16

Work-life integration	Work-life integration coping mechanisms exhausted by personal responsibilities	Prioritisation of time	Personal responsibilities such as work, school, and family are priorities for time and energy expenditure more than personal needs such as PA. Time constraints because of inflexible work hours and family responsibilities leave inadequate personal time to recover from feelings of exhaustion	**3**, 7, **9**, **10**, **11**, 12, 13, 14, 16, 17, 18

Community engagement	Community engagement strategies need to personally relate to people	Conventional marketing of programs has limited impact	Lack of resources, social marketing knowledge, and multilingual skills to communicate about local activities, cause low turn-out and high drop-out rates	**12**, 16
		Lack of tailored activities available to connect with community	Language difficulties and lack of multilingual resources prevent tailoring of activities but friendly nonjudging socialisation gains trust nonetheless	12, 13, **16,** 18

Social support	PA is a social experience	Lack of social support	Social influence is a motivator to engage in PA. Support from community networks including friends, parents, family members, coaches, and health professionals provide both encouragement and sense of security	2, **6**, 8, **10**, **11**, **13**, 14, 15, **16,** 17, 19

		Lacking an exercise companion not a barrier	Some people prefer to exercise alone and not participate in group programs. There are mixed opinions about exercising with co-workers	**3**, **11,** 18, 19

	People might be in isolation because of family culture	Negative social influences	Feeling of guilt as exercise is considered a selfish and low-priority activity by one's own family. In addition, weight-related teasing and social misconceptions between different family cultures impedes social interaction within community	2, 3, 12, **13**, **16,** 17

Psychosocial factors	Low self-esteem makes people feel uncomfortable around peers	Poor self-image	Thinking they are flabby in physical appearance or overweight makes people feel uneasy in fitness clubs with mostly slim and trained people. Body image issues also mean difficulty finding appropriate clothing to wear comfortably in public	2, **11**, **16,** 17, 18

	PA is perceived as a coerced experience	Transport-related walking feels like a chore	Walking is perceived as a negative experience. As a primary form of transport or incidental activity it is described as an exhaustive and burdening necessity	**1**, **2**, 3, 17

		Perceived low personal functioning	Expectation to participate in certain types of exercises might be inappropriate because of advancing age, chronic health conditions, physical disabilities, poor mental health, fatigue, physical discomfort, or current fitness level	**2**, 9, **10**, 13, 17

		However, mental health problems not considered a main barrier	Issues of boredom and stress, being “stuck in a rut” or embarrassed more acknowledged than health problems for lack of exercise	**11, 12**

	People need self-belief through recognised improvement	Perceived low physical competence	Positive experiences of participation such as parental encouragement or “mastery experience” promote ongoing participation, whereas negative experiences discourage participation	**2**, **3,** 18

Bolded indicates key articles most influential in the construct of the summary definition of the first- and second-order constructs.

**Table 2:** COREQ derived quality appraisal.

Quality of individual article	No. articles out of 19	Methodological limitations (no. of articles)
High (14–19)	3	Returning transcripts to study participants for verification (0)

Medium (7–13)	15	Reporting presence of nonparticipants during data collection (1)
		Reporting number of people who refused participation and reasons why (3)

Low (0–6)	1	Providing participants' feedback on the study (3)
		Discussing data saturation (5)
		Providing rationale for number of participants included in the study (5)
		Discussing reflexivity (6)

**Table 3:** Descriptive characteristics of included studies by year of publication.

Source no.	Author	Year	Country	Sample	Ethnicity	Data collection	Topics of interest	Aim
1	Bostock [54]	2000	United Kingdom	*N* = 30 mothers on social security benefits	White (60%), black, Pakistani, Indian, and Gujarati Muslim	Semistructured interviews	Walking, physical fatigue, and psychosocial stress	To contend that “no access to a car” is not only an indicator of low socioeconomic status but of walking as a mode of transport

2	Burton et al. [55].	2003	Australia	*N* = 60 men and women between 18–60 years from three socioeconomic groups (high, middle, and low)	Ethnicity not stated (predominant white assumed)	Semistructured interviews	Recreational physical activity	To explore how influences on recreational physical activity were patterned by socioeconomic position

3	Ball et al. [56]	2006	Australia	*N* = 56 women aged 18–65 years (19 from high, 19 from middle, and 18 from low SES area)	Ethnicity not stated (predominant white assumed)	Semistructured interviews	Physical activity	To investigate why women of low socioeconomic status are less physically active than women of higher SES

4	Bove and Olson [57]	2006	United States	*N* = 28 mothers at least 18 years and one child younger than 12 years. Annual household income less than 200% of the federal poverty level	Ethnicity not stated (predominant white assumed)	In-depth interviews	Physical activity and eating patterns	To understand overweight and obesity from the perspective of low-income mothers living in rural New York state, focusing in particular on challenges to maintaining a healthy weight that might be unique to rural poverty

5	Yen et al. [58]	2006	United States	*N* = 52 women aged 21 to 66 years, at least one child under 18 living at home. From three different neighbourhoods (lower, moderate, and higher income)	Majority Hispanic with non-Hispanic white minority	8 Focus group discussions (FGDs)	Diet, physical activity, and smoking	To investigate women's perceptions of neighbourhood resources and hazards associated with poor diet, physical inactivity, and cigarette smoking

6	Griffin et al. [59]	2007	United States	*N* = 27 adults (70% women and 30% men) living in community where 73% of adult residents have annual income less than $25,000	African Americans	3 FGDs	Physical activity	To increase understanding of how safety and environmental factors influence physical activity among African American residents about how to best design physical activity interventions for their neighbourhood

7	Hartweg and Isabelli-García [60]	2007	United States	*N* = 43 women aged 25 to 61 with family incomes less than 185% of the poverty level	Immigrants from Mexico	7 FGDs	General health, nutrition, and physical activity	To investigate health perceptions of first- and second-generation, low-income, Spanish-speaking women from Mexico and Central America to learn their views of health and also to identify any differences between subcultures

8	Kamphuis et al. [61]	2007	Netherlands	*N* = 38 men and women aged 29–81 years selected based on their neighbourhood's deprivation level and highest educational attainment	Ethnicity not stated (predominant white assumed)	FGDs	Physical activity, fruit, and vegetable consumption	To explore how perceptions of environmental influences on health behaviour pattern across socioeconomic groups in Netherlands

9	Chang et al. [62]	2008	United States	*N* = 80 mothers, 18 to 35 years of age, at least one child enrolled in program providing nutrition consultation for low-income women and children	Non-Hispanic, black or non-Hispanic white	8 FGDs	Healthy eating and physical activity	To identify motivators and barriers to healthful eating and physical activity among low-income overweight and obese non-Hispanic black and non-Hispanic white mothers

10	Bragg et al. [63]	2009	United States	*N* = 50 men and women aged 18–89 years with family income less than $40,000 and *N* = 41 adolescents	African American, Hispanic, non-Hispanic white	12 FGDs	Physical activity	To identify motivators and barriers relative to engagement in physical activity as reported by culturally diverse low-income adolescents and adults

11	Steenhuis et al. [64]	2009	Netherlands	*N* = 27 men and women with lower socioeconomic status using education level as an indicator	Dutch	In-depth interviews	Physical activity and participation in sports activities	To investigate the importance of economic restraints for taking part in sports activities as well as perceptions of low-income people toward different pricing interventions

12	Withall et al. [65]	2009	United Kingdom	*N* = 27 parents aged 16 to 54 years (1 man and 26 women), at least one child under 11 years and living in an economically disadvantaged area	White	5 FGDs	Diet, physical activity, and obesity	To examine reported barriers to consuming a healthy diet and engaging in regular physical activity among low-income families with existing issues of overweight or obesity

13	Kaiser and Baumann [66]	2010	United States	*N* = 20 men and women aged 18 years or over with annual household income less than 200% of Federal Poverty Guidelines for reported household size	Latino and non-Latino	4 FGDs	Physical activity and healthy diet	To describe the perspectives of low-income adults in 2 rural Wisconsin counties on the factors that influence physical activity and healthy eating

14	Greaney et al. [67]	2012	United States	*N* = 35 adults aged 18–45 years (20 women, 15 men). Majority report household income of less than $20,000 and not having graduated from high school	Immigrants from South America, Central America, Mexico, or the Caribbean	4 FGDs	Diet and physical activity	To explore how migration influenced physical activity and dietary behaviours among Latino immigrants

15	Hartweg et al. [68]	2012	United States	*N* = 30 women approximately, aged 18–64 years with family income less than 185% of United States poverty level	Immigrants from Mexico	5 FGDs	Physical activity	To elicit recent Mexican immigrant women's perceptions of “being physically active” and to describe how living in United States has influenced their perceptions of being physically active

16	Mansfield et al. [69]	2012	Canada	*N* = 42 mothers (median age 35.7 years, standard deviation 7.7 years) self-identified as SED using MacArthur Scale of Subjective Social Status, have at least one child ≤14 years of age still living at home	Multiethnic	6 FGDs	Physical activity	To identify the individual, social, and environmental factors influencing utilitarian and leisure time physical activities of multiethnic socioeconomically disadvantaged mothers

17	Mohamed et al. [70]	2014	United States	*N* = 20, Somali men living in Rochester, Minnesota. Age ranged from 24 to 65	Somali men	3 FGDs and 3 in-depth interviews	Physical activity	Determine perceptions of physical activity

18	Wieland et al. [71]	2015	United States	*N* = 127 (adults 54 and adolescents 73), immigrants and refugees mean annual income varied from USD 14,862 to 24,857	Immigrants and refugees of Cambodian, Mexican, Somali, and Sudanese background	16 FGDs	Physical activity	Determine reasons for low level of PA among the immigrants and refugees

19	Gray et al. [72]	2016	United Kingdom	*N* = 28 among older adults with low/high SES, using self-determination theory and self-efficacy theory framework	Older adults with low/high SES	4 FGDs	Physical activity	Explore motives and barriers to physical activity among older adults of differing socioeconomic status
